# mRNA vaccination of rabbits alters the fecundity, but not the attachment, of adult *Ixodes scapularis*

**DOI:** 10.1038/s41598-023-50389-6

**Published:** 2024-01-04

**Authors:** Jaqueline Matias, Yingjun Cui, Geoffrey E. Lynn, Kathleen DePonte, Emily Mesquita, Hiromi Muramatsu, Mohamad G. Alameh, Garima Dwivedi, Ying K. Tam, Norbert Pardi, Drew Weissman, Erol Fikrig

**Affiliations:** 1 Section of Infectious Diseases, Department of Internal Medicine, School of Medicine, Yale University, New Haven, CT 06520 USA; 2 Department of Microbiology, Perelman School of Medicine, University of Pennsylvania, Philadelphia, PA 19104 USA; 3 Department of Medicine, Perelman School of Medicine, University of Pennsylvania, Philadelphia, PA 19104 USA; 4 Acuitas Therapeutics, Vancouver, BC V6T 1Z3 Canada

**Keywords:** Immunology, Molecular biology

## Abstract

19ISP is a nucleoside-modified mRNA-lipid nanoparticle vaccine that targets 19 *Ixodes scapularis* proteins. We demonstrate that adult *I*. *scapularis* have impaired fecundity when allowed to engorge on 19ISP-immunized rabbits. 19ISP, therefore, has the potential to interrupt the tick reproductive cycle, without triggering some of the other effects associated with acquired tick resistance. This may lead to the development of new strategies to reduce *I. scapularis* populations in endemic areas.

## Introduction

Tick saliva is composed of a diverse set of bioactive molecules including proteins that facilitate tick feeding^1,2^. Some of these proteins have been associated with host immune responses that lead to acquired tick resistance (ATR)^3^. It was recently shown that a lipid nanoparticle containing the nucleoside-modified mRNAs encoding 19 tick salivary proteins, named 19ISP, can induce ATR. Guinea pigs immunized with 19ISP, and then exposed to *Ixodes scapularis* nymphs developed robust *erythema* at the tick bite sites, diminished tick engorgement, and early tick detachment^4^. Guinea pigs, however, are not a good laboratory model for adult *I. scapularis* feeding, molting and fecundity, which prevents a complete assessment of the influence of 19ISP on this stage of the tick life cycle.

ATR has also been observed in rabbits, dog, and cattle^5–7^, and rabbits are an excellent animal laboratory model for studying feeding by adult *I. scapularis*. We, therefore, tested the ability of 19ISP to induce ATR against adult *Ixodes scapularis* in rabbits with a focus on the ability of 19ISP to impact fecundity.

**Figure 1:**
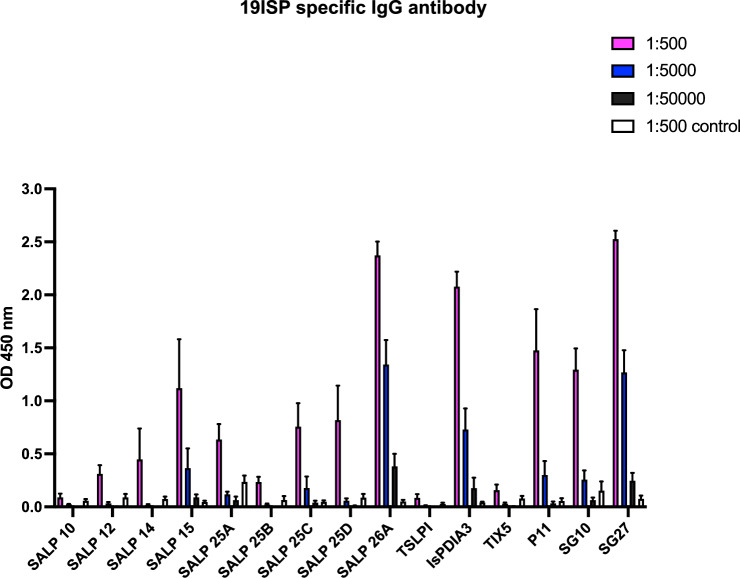
19ISP immunizations elicit antibody responses in rabbits. Rabbits were immunized intradermally with 19ISP (n = 6) or control Luc mRNA-LNP (n = 6). Two weeks after the last immunization, sera were collected to assess the humoral response. Specific IgG antibodies were detected by ELISA using an optical density (OD) of 450 nm. The error bars represent mean ± SEM.

Rabbits were immunized with 19ISP, or a lipid nanoparticle containing firefly luciferase mRNA as a control and exposed to adult *I. scapularis*, in order to determine whether vaccination could interfere with the reproductive fitness of the ticks. The rabbits received 3 doses of 19ISP or the control formulation, given at 4-week intervals. Two weeks after the last boost, sera were obtained from the animals, and examined by ELISA for antibodies to antigens within 19ISP, at dilutions of 1:500, 1:5000 and 1:50,000. Rabbits elicited antibodies against 11 of the recombinant proteins tested (Salp12, Salp14, Salp15, Salp25A, Salp25C, Salp25D, Salp26A, IsPDIA3, P11, SG10, SG27) (Fig. 1). The rabbits did not show antibodies against TSLPI and TIX, which differs from the results observed when guinea pigs were immunized with the same batch of 19ISP vaccine^4^. The dose administered in rabbits was calculated in a similar way to that administered to guinea pigs^4^, based on the weight of the animals. When comparing antibody responses between rabbits and guinea pigs, the rabbits were generally shown to be less responsive to immunization with 19ISP^4^ . The rabbits also did not show a strong antibody response to Salp14 compared to guinea pigs immunized with 19ISP^4^, and this may have been one of the reasons for the absence of *erythema* in the rabbits. Salp14 was the antigen most commonly associated with *erythema* in guinea pigs exposed to *I. scapularis* nymphs, when all the 19 components within 19ISP were analyzed, either individually or in smaller combinations^8,9^.

**Figure 2:**
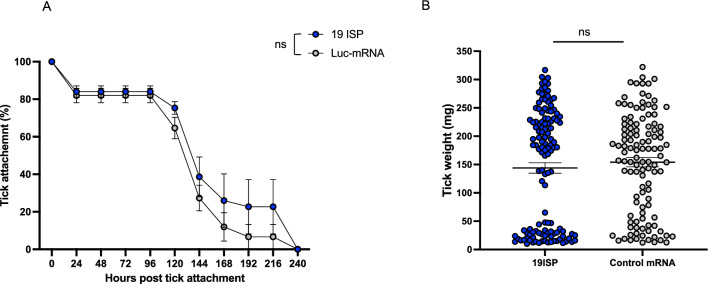
Tick feeding kinetics on rabbits immunized with 19ISP. Rabbits (n = 6 per group) were immunized three times with 19ISP or a control Luc mRNA-LNP and challenged with 50 adults *I*. *scapularis*. The animals were monitored to assess tick detachment, recovery, and female engorgement. The graph shows the percentage of ticks that remain attached, and the detachment at a given time point (**A**). The success of tick feeding was determined by examining engorgement weights of the recovered ticks (**B**). Statistical significance was assessed using a nonparametric Mann–Whitney test (ns, *P* > 0.05).

One week after collecting the blood sample, the rabbits were exposed to adult *I. scapularis*. The ticks on the animals were monitored daily for attachment (Fig. 2A), and the weights of the collected ticks were determined (Fig. 2B). No significant differences were observed in the duration of time that *I. scapularis* remained attached to I9ISP-immunized or control rabbits. The engorgement weights of the ticks that fed on both groups of animals was also similar and no difference in *erythema* at the bite site was observed between the groups. These results are different from those observed in guinea pigs with nymphal *I. scapularis*^4,8^. In guinea pigs, early detachment or rejection of the nymphs was observed by 72 h. This influence of 19ISP mRNA immunization on the feeding time of nymphs, also resulted in a marked reduction of the weights of the recovered ticks. These data underscore the differences observed in rabbits using adult *I. scapularis*.

**Figure 3:**
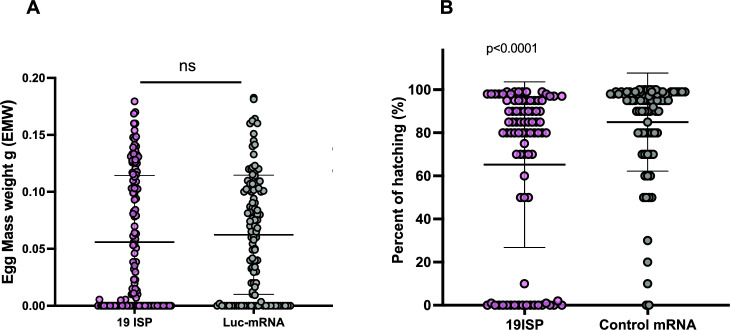
Biological parameters of *Ixodes scapularis* after feeding on 19ISP-immunized rabbits. Adult ticks were collected after detachment, weighed, and separated individually. The graph shows the weight of the egg mass produced by each tick (**A**). The hatching percentage of each egg mass was obtained from the visual count of larvae (**B**) and statistical analysis was performed using a nonparametric Mann Whitney test, P value < 0.0001. The error bars represent mean with SD.

To assess fecundity, all ticks were evaluated for oviposition regardless of engorgement weight. Ticks were collected, stored individually, and allowed to complete their reproductive cycle. The weight of the egg mass generated by each female (Fig. 3A) was similar in ticks that fed on 19ISP-immunized or control rabbits. Females from both groups who did not posture or had a very low weight were also included as a part of Fig. 3A. Interestingly, a significant difference was observed in the percentage of eggs that hatched into larvae between *I. scapularis* that fed on 19ISP-immunized rabbits compared to the ticks that fed on control animals (Fig. 3B). These data suggest that the 19ISP vaccination can significantly impair a major parameter of *I. scapularis* reproduction, without influencing some of the other parameters associated with ATR.

Our study demonstrates that 19ISP immunization can significantly interfere with the ability of adult *I. scapularis* to successfully complete their reproductive cycle. In particular, the capacity of adults to produce viable larvae was substantially reduced. Interestingly, some differences in ATR were evident upon 19ISP immunization in guinea pigs and rabbits. An immune response to Salp14 in guinea pigs is linked to *erythema* at the tick bite site. Surprisingly, rabbits do not develop robust antibodies to Salp14 following 19ISP immunization, and these animals did not develop significant *erythema* at the tick bite site. These data underscore the importance of the different host immune response to specific tick proteins in the development of selected aspects of ATR. These data also help expand the principle that not all aspects of ATR, including *erythema*, tick engorgement and attachment and tick fecundity occur via the same mechanisms, or are associated with host responses to the same antigens.

Understanding the specific host responses associated with ATR is complex and requires the combination of different techniques. A detailed analysis of the bite site, including the examination of specific cellular responses using single cell RNA seq, as well as an examination of local innate, cellular and metabolic responses will help to guide future research. Adult *I. scapularis* commonly feed on deer to complete their life cycle, and it will be important to determine if the alterations in fecundity observed in the rabbit model extend to deer. Overall, these studies suggest that 19ISP can interfere with the fecundity of adult *I. scapularis*, and this finding opens new strategies to reduce tick populations in endemic areas.

## Methods

### Ethics

All experiments were conducted in accordance with the Guide for the Care and Use of Laboratory Animals of the National Institutes of Health, USA. The animal protocols were approved by the Yale University Institutional Animal Care and Use Committee (YUIACUC—protocol number 2023-07941). The authors complied with the ARRIVE guidelines.

### Ticks, animals and immunization

*I. scapularis* adults were obtained from the Oklahoma State University (Stillwater, OK) and maintained in an incubator at 23 °C and 90% relative humidity under a 14 h light, 10 h dark photoperiod. Female and male ticks were kept together for at least 48 h before challenge under the conditions mentioned above. New Zealand white rabbits (Charles River) were used for immunizations and tick challenge experiments.

Rabbits were immunized intradermally with 200 μg of 19ISP (n = 2) or luciferase mRNA as a control (n = 2). The animals were boosted twice at 4-week intervals. Three weeks after the last booster, rabbits were sedated with acepromazine (0.75 mg/kg) and 25 female and 25 male adult ticks were placed on the animal’s ears. Rabbits were monitored daily for evidence of tick rejection, recovery, and *erythema* at the bite site. The experiment was repeated three times, totaling 6 animals per group.

In addition, all the rabbits wore Elizabethan collars and the ear that received the tick was isolated with an adapted sock, which prevented the ticks from moving to another location. The animals were prevented from self-cleaning and received a daily dose of 1.25 mg/kg diazepam as a veterinary recommendation to reduce stress. During tick feeding, rabbits were housed individually in closed cages with wire grates suspended over water. The ticks that attached to the rabbits were allowed to detach naturally.

All recovered fed ticks were kept individually in plastic tubes sealed and maintained in an incubator under the conditions mentioned above until the end of egg laying (around 6 weeks). The egg mass was weighed and kept in the incubator until the hatching of the larvae (8 weeks). The hatching success was obtained by the value of visual count of larvae.

### Laboratory methods

To assess antigen-specific antibody responses, ELISA was performed as described previously^4^. Briefly, 96-well ELISA plates were coated overnight with 250 ng of recombinant protein diluted in carbonate-bicarbonate buffer pH 9.6, washed with PBST (PBS with 0.05% Tween 20) and blocked with 3% bovine serum albumin for 1 h at 37° C. Rabbit sera were serially diluted (1:500, 1:5000, or 1:50,000) and incubated for 2 h at 37° C. Wells were washed, and reactivity was detected using goat anti-rabbit IgG-HRP and TMB HRP substrate solution (Thermo Fisher Scientific). TMB stop solution was added and the plates were read at 450 nm.

mRNA-lipid nanoparticles (LNPs) were formulated as previously described^10^ and this LNP contains 19 mRNAs encoding individual *I. scapularis* salivary proteins or firefly luciferase (Luc) mRNA^4^. mRNAs were transcribed from a linearized plasmid DNA template with a 101 nucleotide-long poly(A) tail and instead of UTP, N-1-methylpseudouridine (m1Ψ) 5’-triphosphate (TriLink) was used to generate modified nucleoside-containing mRNA. During the in vitro transcription, co-transcriptional capping was performed using the trinucleotide cap1 analog, CleanCap (TriLink). mRNAs were purified by cellulose purification, as described^11^, analyzed by gel electrophoresis and frozen at −20 °C. All mRNAs were combined and then encapsulated using an aqueous solution of mRNA at pH 4.0 and mixed with a solution of lipids dissolved in ethanol^12,13^. The solution contains an ionizable cationic lipid/ phosphatidylcholine/ cholesterol/ polyethylene glycol (PEG) lipid (proprietary of Acuitas, Vancouver, Canada) (50:10:38.5:1.5 mol/mol). RNA was mixed with the lipids at a ratio of ~ 0.05 (wt./wt.), LNP had a diameter of ~ 80 nm as measured by dynamic light scattering using a Zetasizer Nano ZS (Malvern Instruments Ltd, Malvern, UK) instrument, and stored at − 80 °C.

### Statistical analyses

Data were analyzed using Prism 9.5.1 software (GraphPad Software, CA). Data are represented as the mean ± standard error of the mean (SEM) or mean with standard deviation (SD). The significance of the difference between control and experimental group was determined by Mann Whitney test. P ≤ 0.05 was considered statistically significant.

## Data Availability

The data that support the findings of this study are available on request from the corresponding authors J.M and E.F.
